# Synergistic effect of eugenol with Colistin against clinical isolated Colistin-resistant *Escherichia coli* strains

**DOI:** 10.1186/s13756-018-0303-7

**Published:** 2018-01-29

**Authors:** Yi-ming Wang, Ling-cong Kong, Jie Liu, Hong-xia Ma

**Affiliations:** College of Animal Science and Technology, Jilin Agricultural University, Xincheng Street No.2888, Changchun, 130118 China

**Keywords:** eugenol, colistin-resistant *Escherichia coli*, mcr-1 gene, molecular docking

## Abstract

**Background:**

Bacterial infections have become more challenging to treat due to the emergence of multidrug-resistant pathogenic bacteria. Combined antibiotics prove to be a relatively effective method to control such resistant strains. This study aim to investigate synergistic activity of eugenol combined with colistin against a collection of clinical isolated *Escherichia coli* (*E.coli*) strains, and to evaluate potential interaction.

**Methods:**

Antimicrobial susceptibility, minimum inhibitory concentration (MIC) and fractional inhibitory concentration index (FICI) of the bacteria were determined by disk diffusion assay, broth microdilution method and checkerboard assay, respectively. The mcr-1 mRNA expression was measured by Real-time PCR. To predict possible interactions between eugenol and MCR-1, molecular docking assay was taken.

**Results:**

For total fourteen strains including eight colistin-resistant strains, eugenol was determined with MIC values of 4 to 8 μg/mL. Checkerboard dilution test suggested that eugenol exhibited synergistic activity when combined with colistin (FICI ranging from 0.375 to 0.625). Comparison analysis of Real-time PCR showed that synergy could significantly down-regulate expression of mcr-1 gene. A metal ion coordination bond with catalytic zinc atom and a hydrogen bond with crucial amino acid residue Ser284 of MCR-1 were observed after molecular docking, indicating antibacterial activity and direct molecular interactions of eugenol with MCR-1 protein.

**Conclusions:**

Our results demonstrated that eugenol exhibited synergistic effect with colistin and enhanced its antimicrobial activity. This might further contribute to the antibacterial actions against colistin-resistant *E.coli* strains.

**Graphical abstract:**

Synergistic effect of eugenol with colistin against colistin-resistant Escherichia coli isolates.
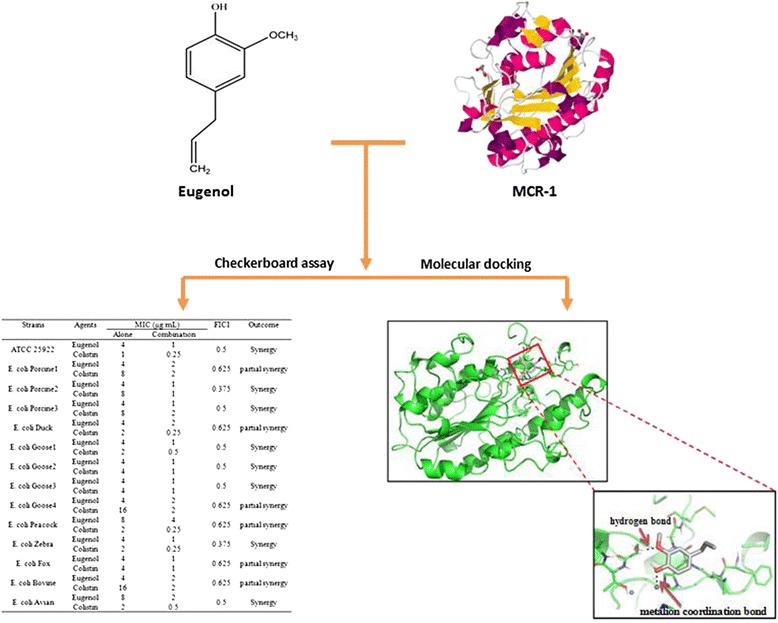

## Background

The challenge presented by the emergence of antibiotic resistance is increasingly significant. Colistin, as an old member of polymycin group, has a better broad-spectrum activity against Gram-negative bacteria including most species of Enterobacteriaceae and is regarded as the last resort for treatment of multiple drug resistance (MDR). However, the currently emerged plasmid-mediated colistin resistance mechanism MCR-1 has already been reported, which has posed great threats to both human and animals [1–4]. Therefore, it is urgently needed to find effective combination therapy and antibiotic succedaneum to eradicate the colistin-resistant bacteria and to slow down its spread and prevalence.

Essential oils (EOs) are naturally derived from many plants with a series of pharmacological activities and have been widely used in traditional medicine and food preservation. Many studies have documented EOs to be effective antimicrobial against *E. coli*, *S. aureus*, *Lactobacillus sp.*, *Bacillus thermoacidurans*, *Salmonella sp.* [5–9]. Moreover, the synergistic combinations with other EOs and conventional antimicrobials have also been widely reported since last decade [10–14]. Eugenol, a phenylpropanoid found in many plants and the major active essential oil component of clove, has been reported to have antioxidant properties, protective effect on the cardiovascular system, antibacterial and antifungal effect in previous studies. Several studies support that its antibacterial activity is closely related to the capability to permeabilize the cell membrane, destroy membrane integrity and facilitate the entry of eugenol into the cytoplasm which finally interacts with proteins and enzymes, and leads to the leakage of intracellular substances [10, 11, 15–17].

The current study aim to investigate the synergistic activity of eugenol combined with colistin against a collection of clinical *E.coli* isolates and to predict possible interactions between eugenol and MCR-1.

## Methods

### Antimicrobial agents and culture medium

Eugenol was obtained from Macklin Biochemical (Shanghai, China) Co.,Ltd. and was dissolved in MH broth. Dimethyl sulfoxide (DMSO, Sigma–Aldrich, USA) was added with a final concentration of 1%. Triphenyltetrazolium chloride (TTC) was purchased from Biotopped Co., LTD (Beijing, China). Colistin was purchased from National Institutes for Food and Drug Control (Beijing, China). Mueller–Hinton broth (MHB) and Mueller–Hinton agar (MHA) were obtained from Qingdao Hope Bio-Technology Co., LTD (Qingdao, China). Lysogeny broth (LB) from Sangon Biotech (Shanghai, China) Co., Ltd. Commercial antibiotic paper disks: AMP(Ampicillin,10 μg), AZM(Azithromycin,15 μg), CFZ(Cefazolin,30 μg), CTX(Cefotaxime,30 μg), DOX(Doxycycline,30 μg), FF(Florfenicol,30 μg), FRZ(furazolidone,300 μg), GEN(gentamicin,10 μg), KAN(kanamycin,30 μg), LVX(levofloxacin,5 μg), NEO(Neomycin,30 μg), NOR(Norfloxacin,10 μg), POL(PolymyxinB,300 IU), STR(Streptomycin,10 μg), TCY(Tetracycline,30 μg) were purchased from Hangzhou Microbial Reagent Co.,Ltd. (Hangzhou, China).

### Preparation of the McFarland standard

0.5 ml of 1.17% BaCl_2_ (*w*/*v*) was added to 99.5 ml of 0.18 M H_2_SO_4_ (1%*v*/v) with constant stirring.

### Bacterial strains

Thirteen clinical isolated *E.coli* strains were collected from duck (*n* = 1), peacock (n = 1), bovine (n = 1), fox (n = 1), zebra (n = 1), porcine (*n* = 3), goose (*n* = 4) and avian (n = 1). All porcine isolates, two goose isolates (*E. coli* Goose 3, *E. coli* Goose 4) and one bovine isolate were previously confirmed to be mcr-1 gene positive according to the PCR test. *E.coli* ATCC 25922 was employed in this study as quality control strains.

### Disk diffusion assay

The drug resistance status of twelve clinical isolated *E. coli* strains was determined using agar disk diffusion method. Briefly, the inoculum was adjusted to 0.5 McFarland standards (equivalent to 10^8^cfu/ml). Steriled plates containing MH agar medium were seeded with 100 μl each bacterial strain. Fifteen kinds of commercial antibiotic paper disks were placed on the surface of the inoculated plates. Incubations were carried out for 16 h at 37 °C. Antibacterial susceptibility was determined by measuring the diameter of the inhibition zone (in mm) generated around the disc according to the manufacturer’s protocol. Discs of cefazolin were used as positive controls. All the tests were performed in triplicate.

### Determination of MIC

The MIC was determined by broth microdilution method [18]. Bacterial colonies of each strain were cultured in LB broth at 37 °C to reach McFarland standards 0.5. The suspensions were further diluted to obtain an inoculum of 10^6^ cfu/ml. The drugs, serial 2-fold diluted in MHB, were inoculated with bacterial suspension to obtain a final concentration of 5 × 10^5^ cfu/ml. Then, TTC was added as growth indicator of less than 2% of the total test volume. All strains were determined antibiotics resistance according to MIC breakpoint of colistin (EUCAST, Version 7.1). MICs were defined as the lowest concentration of test samples that resulted in a complete inhibition of visible growth after incubation.

### Time-kill curves

Time-kill methods were used to evaluate the antibacterial effects of eugenol against all *E. coli* strains by measuring the reduction in the number of cfu/mL within 160 min. Eugenol (corresponding to MIC)was incubated with equal volume *E. coli* strains. For control, MHB was added instead of eugenol. All samples were incubated at 37 °C. After 0, 20, 40, 80, 120, 160 min of incubation, 100 μl samples were removed, 10-fold diluted and spread on the surface of MHA for colony counting. Each assay was performed in triplicate.

### Checkerboard assay

The combined antibacterial effects of eugenol with colistin were assessed by checkerboard test as previously described [19]. Briefly, both eugenol and colistin were diluted to make seven gradient concentrations: from 1/16 MIC to 2 MIC. Each longitudinal column tubes containing same concentration of drug A, and each horizontal row of tubes containing same concentration of drug B. Each tube was inoculated with bacterial suspension to make a final concentration of approximately 5 × 10^5^ CFU/ml. Moreover, single drug control tubes, colistin-free control tubes, eugenol-free control tubes and blank control tubes were also set. *E. coli* ATCC 25922 was used as sensitivity control strain. All tubes were incubated at 37 °C for 16 h under aerobic conditions. The experiment was repeated in triplicate. MIC values obtained for a given combination were used to evaluate the effects of combination between eugenol and colistin by calculating the FICI using formula: FICI = MIC of eugenol in combination/MIC of eugenol alone; FIC of colistin = MIC of colistin in combination/MIC of colistin alone. “Synergy” was defined when FICI≤0.5; 0.5 < FICI≤0.75 means “partial synergy”; 0.76 < FICI≤1 denotes “additive”; 1 < FICI≤4 denotes “indifferent”; while “antagonistic” in cases which the FIC index > 4. In this study, synergy and partial synergy were defined as synergy relationship, while additive, indifferent and antagonistic were regarded as non-synergy.

### Real-time PCR

Expression of mcr-1 gene at mRNA level was also evaluated using Real-time PCR. Bacteria was cultured to logarithmic phase, eugenol was then mixed with bacteria to make a final concentration of sub-MIC (1/4 MIC). While in control group, MH broth was added instead of eugenol. The mixture was incubated at 37 °C and shaking at 160 rpm for 16 h. Total RNA was extracted using RNAiso Plus (TaKaRa) and was then reverse transcribed to cDNA using PrimeScript ™ RT reagent Kit (TaKaRa). The extracted RNA was adjusted the same concentration during DNA elimination process before reverse transcription.

Real-time PCR was carried out in an Applied Biosystems 7500 Real-Time PCR System (USA). The primers were retrieved and designed according to *E. coli* strain SHP45 plasmid pHNSHP45 complete sequence (GenBank accession no. KP347127.1). The amplifications were performed in 20 μl reaction mixtures containing 10 μl mastermix with SYBR ® Green I, 0.4 μl ROX Reference Dye II(SYBR Premix Ex Taq II, Tli RNaseH Plus, Code No.RR820A/B, Takara), 0.8 μl mcr-1 forward and reverse primer (10 μM), 6.0 μl double distilled H_2_O, respectively, and 2.0 μl template were added to each reaction sample. 16SrRNA of the strain was used as a reference gene (Table 1).

**Table 1:** Sequences of primers used in this study

Primer name	Sequence(5′-3′)	Product size(bp)
mcr-1-F	TGCTCCAAAATGCCCTACAGACC	141
mcr-1-R	TGCCCCAAGTCGGATAATCCAC	
16SrRNA-F	TGTCGTCAGCTCGTGTTGTG	130
16SrRNA-R	ATCCCCACCTTCCTCCAGTT	

The reacting condition was set as two steps method as follows: pre-denaturation at 95 °C for 30s, 40 cycles consisting of denaturation at 95 °C for 5 s, annealing at 60 °C for 34 s. All templates were run in triplicate.

### Molecular docking

To study the possible interaction between eugenol and MCR-1, molecular docking was performed via AutoDock Vina software. Crystal structure of MCR-1 (PDB ID: 5GRR), available at Protein Data Bank was obtained in PDB format. Weighting parameters of scoring function include spatial interaction, hydrophobic interaction, hydrogen-bonding energy and number of rotatable keys in ligand. Affinity was measured to assess docking. The lower the parameter is, the ligand is more likely to bind with the active pocket. PyMol molecular graphics system was used for analysis of their modes of interaction with binding site residues.

### Statistical analysis

Data were expressed as mean ± standard deviation. Statistical significance was analyzed by one-way analysis of variance (ANOVA) using SPSS 17.0 software. In all comparisons, *P < 0.01* or *P < 0.05* was considered statistically significant.

## Results

### Antimicrobial susceptibility test

According to the obtained results, the thirteen clinical *E. coli* isolates were resistant to selected antibiotics to varying degrees. Details of drug resistance status are shown in Tables 2 and 3, which clearly demonstrated that all isolates were multi-drug resistant *E. coli*.

**Table 2:** Antimicrobial resistance profile of clinical *Escherichia coli* isolates

Strains	Isolates used	Antimicrobial resistance^a^
*E. coli* Porcine1	colistin-resistant	STR, TCY
E. coli Porcine2	colistin-resistant	AMP, FF, GEN, KAN, TCY
E. coli Porcine3	colistin-resistant	AMP, CFZ, CTX, FF, GEN, KAN, LVX, STR, NEO, NOR, TCY
E. coli Duck	colistin-sensitive	AMP, DOX, FRZ, KAN, STR, TCY
E. coli Goose1	colistin-sensitive	AMP, DOX, FRZ, GEN, KAN, LVX, STR, NEO, NOR, TCY
E. coli Goose2	colistin-resistant	AMP, AZM, DOX, FRZ, TCY
E. coli Goose3	colistin-resistant	AMP, CFZ, DOX, FRZ, LVX, NOR, STR, TCY
E. coli Goose4	colistin-resistant	AMP, AZM, DOX, FRZ, GEN, KAN, LVX, NOR, STR, NEO, TCY
E. coli Peacock	colistin-sensitive	AMP, AZM, DOX, GEN, LVX, NOR,STR, TCY
E. coli Zebra	colistin-sensitive	AMP, AZM, CFZ, CTX, DOX, FF, LVX, NOR,STR, TCY
E. coli Fox	colistin- resistant	AMP, AZM, CFZ, DOX, FRZ, GEN, LVX, NOR, STR, NEO, TCY
E. coli Bovine	colistin- resistant	AMP, FRZ, KAN, STR
E. coli Avian	colistin-sensitive	AMP, FRZ, GEN, KAN, STR

^a^The strain is resistant to the antibiotics

**Table 3:** MIC of antibiotics against *Escherichia coli* strains MIC

Antibiotics	AMP	AZM	CFZ	CTX	DOX	FF	GEN	KAN	LVX	NEO	NOR	STR	TCY
Strains													
ATCC 25922	4	8	1	< 1	2	2	< 1	2	0.5	4	1	8	1
E.coli Porcine1	16	8	2	< 1	4	4	16	16	2	4	4	64	16
E.coli Porcine2	128	8	4	≤1	4	8	128	128	2	8	4	64	16
E.coli Porcine3	256	8	4	16	8	> 8	128	256	16	> 64	32	64	32
E. coli Duck	128	8	4	< 1	32	2	32	64	2	8	4	128	64
E.coli Goose1	> 256	16	4	≤1	128	8	256	32	16	> 64	32	128	256
E.coli Goose2	32	8	4	< 1	32	4	128	128	2	8	4	64	128
E.coli Goose3	> 256	8	16	1	128	8	256	256	64	64	> 64	128	256
E.coli Goose4	> 256	32	4	1	128	> 8	64	512	32	> 64	64	128	256
E.coli Peacock	> 256	8	4	1	> 256	8	256	512	32	8	> 64	256	> 512
E. coli Zebra	16	8	> 32	> 32	32	8	128	32	8	8	16	256	128
E. coli Fox	> 256	32	32	1	> 256	> 8	128	32	32	> 64	> 64	64	> 512
E. coli Bovine	16	8	2	< 1	4	2	32	32	2	8	4	32	4
E. coli Avian	> 256	8	2	< 1	16	2	128	32	2	8	4	64	16

### Time-kill analysis of eugenol

**Fig. 1:**
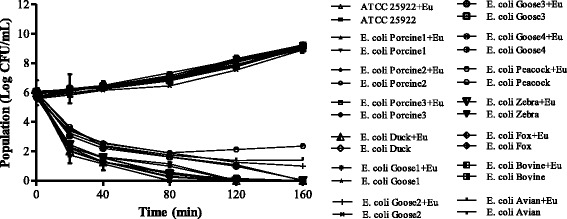
Effect of eugenol(control and MIC) on the viability of *E. coli* strains

Based on the results of MIC assay, the time-kill curve was used to describe the viability after treated with eugenol. As shown in Fig. 1, although at MIC, eugenol exhibited a bactericidal effect on most *E. coli* strains within 120 min. In contrast, eugenol only inhibited the growth of *E. coli* Goose 2, *E. coli* Peacock and *E. coli* Avian rather than inactivation. Similarly, the MIC value often met the MBC value in our previous study, which indicated that eugenol may act as a bactericidal agent. The above results suggested that eugenol exerted a strong and rapid antibacterial effect on *E. coli.*

### Synergistic effect of eugenol with colistin

Eugenol was evaluated for its antimicrobial activity against thirteen clinical isolated *E. coli* strains and one sensitivity control strain. The results of the antimicrobial activity showed that eugenol presented almost identical antimicrobial activity against all tested strains (MIC, 4 to 8 μg/mL). As shown in Table 4, for *E. coli* isolates *E. coli* Porcine 2 and *E. coli* Zebra, the combination of eugenol and colistin exerted strong synergistic effect, where the addition of eugenol at 1/4MIC concentrations resulted in 8-fold MIC reduction of colistin (FICI = 0.375, respectively). For ATCC 25922, *E. coli* Porcine 3, *E. coli* Goose 1, *E. coli* Goose 2, *E. coli* Goose 3 and *E. coli* Avian, 4-fold MIC reduction of colistin was observed after combined with 1/4MIC eugenol (FICI = 0.5). For *E. coli* Porcine 1, *E. coli* Duck, *E. coli* Goose 4, *E. coli* Peacock, *E. coli* Fox and *E. coli* Bovine, the addition of 1/4 to 1/2 MIC eugenol could partially synergize with colistin and resulted in 2 to 8-fold MIC reduction (FICI = 0.625).

**Table 4:** MIC and FICI of eugenol and colistin against *Escherichia coli* strains

Strains	Agents	MIC (μg/mL)		FICI	Outcome
		Alone	Combination		
ATCC 25922	Eugenol	4	1	0.5	Synergy
	Colistin	1	0.25		
E. coli Porcine1	Eugenol	4	2	0.625	partial synergy
	Colistin	8	2		
E. coli Porcine2	Eugenol	4	1	0.375	Synergy
	Colistin	8	1		
E. coli Porcine3	Eugenol	4	1	0.5	Synergy
	Colistin	8	2		
E. coli Duck	Eugenol	4	2	0.625	partial synergy
	Colistin	2	0.25		
E. coli Goose1	Eugenol	4	1	0.5	Synergy
	Colistin	2	0.5		
E. coli Goose2	Eugenol	4	1	0.5	Synergy
	Colistin	4	1		
E. coli Goose3	Eugenol	4	1	0.5	Synergy
	Colistin	4	1		
E. coli Goose4	Eugenol	4	2	0.625	partial synergy
	Colistin	16	2		
E. coli Peacock	Eugenol	8	4	0.625	partial synergy
	Colistin	2	0.25		
E. coli Zebra	Eugenol	4	1	0.375	Synergy
	Colistin	2	0.25		
E. coli Fox	Eugenol	4	1	0.625	partial synergy
	Colistin	4	1		
E. coli Bovine	Eugenol	4	2	0.625	partial synergy
	Colistin	16	2		
E. coli Avian	Eugenol	8	2	0.5	Synergy
	Colistin	2	0.5		

### Analysis of real-time PCR

**Fig. 2:**
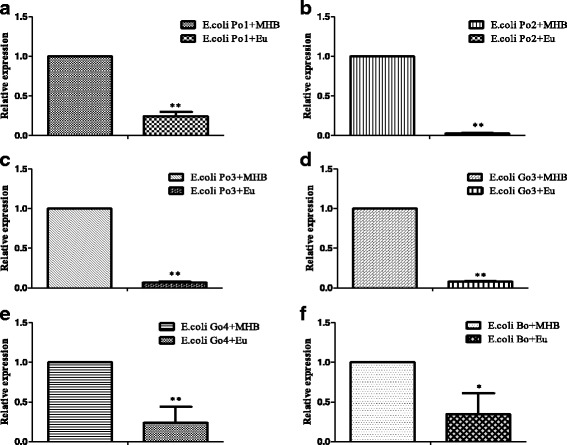
Relative expression of mcr-1 gene. E. coli Po1 + MHB, E. coli Po1 + Eugenol (**a**), E. coli Po2 + MHB, E. coli Po2 + Eugenol (**b**), E. coli Po3 + MHB, E. coli Po3 + Eugenol (**c**), E. coli Go3 + MHB, E. coli Go3 + Eugenol (**d**), E. coli Go4 + MHB, E. coli Go4 + Eugenol (**e**), E. coli Bo+MHB, E. coli Bo+Eugenol (**f**). All data were expressed as mean ± S.D.,*n* = 3. ** *P* < 0.01, * *P* < 0.05 vs. non-synergy group

To investigate if the synergy of eugenol with colistin has an effect on drug resistant gene at mRNA level, relative expression of mcr-1 were compared between synergy group and non-synergy group. Referring to the results presented in Fig. 2, significant differences of mcr-1 gene relative expression can be seen among synergy group and non-synergy groups, which clearly indicating that colistin resistance gene mcr-1 was down-regulated by additional eugenol. Therefore, we speculate that eugenol might exhibits a direct inhibiting effect on drug resistant gene.

### Docking study of eugenol and MCR-1

**Fig. 3:**
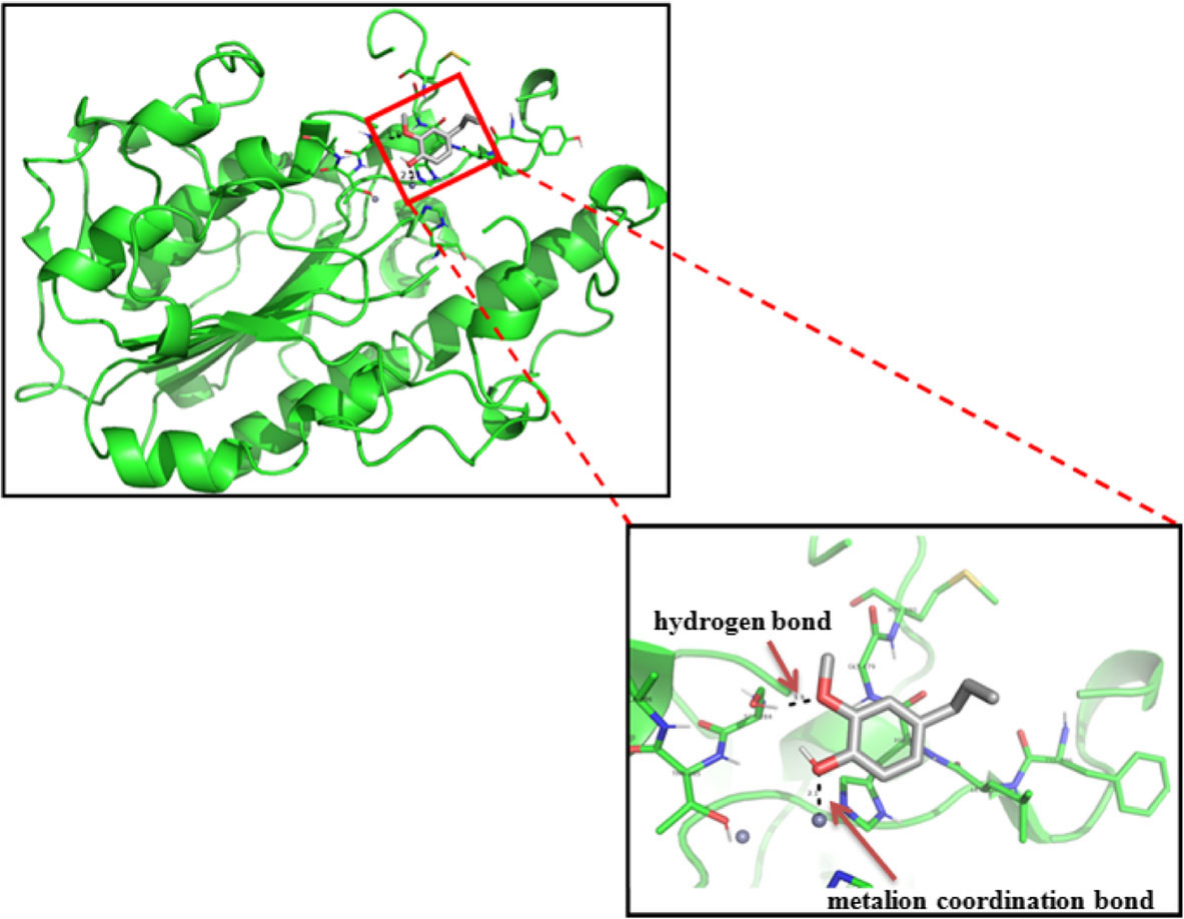
Putative pattern of interaction between eugenol and MCR-1 protein. The structure eugenol was shown in gray, the green stick around eugenol are amino acid residue. Carbon atoms were shown in gray, hydrogen atoms in white, oxygen atoms in red, nitrogen atoms in blue, sulphur atoms in gamboges

Referring to the results presented in Fig. 3, optimal interaction and relative affinity parameters were used to elucidate the spatial conformation among the MCR-1 protein and bioactive group of eugenol after docking. The results showed that free energy of binding was − 10.087 kcal/mol, which indicating a possible bind between MCR-1 protein and eugenol. A total of 24 ligand (eugenol) atoms could dock with MCR-1, and 2 among them were flexible. Hydrogen bonding (Hbond), important in determining the structure and function of a biological molecule, possessed a score of − 2.07393. A metal ion coordination bond (2.1 Å length) between phenolic hydroxyl group in eugenol and zinc atom was observed. Moreover, methoxy in ortho-position could bind with Ser284 in the form of hydrogen bond with a distance of 1.9 Å.

## Discussion

The emergence of MDR bacterial strains appear to be the major cause of animal bacterial disease and have made commonly used therapy invalid, which have caused great economic loss and also posed great threats to human and animal health. Polymyxins consist of polymyxins A to E, of which polymyxin B and polymyxin E (colistin) are commonly used in clinical mainly against the Gram-negatives [20]. The class of polymyxins is considered as the last option against MDR that are resistant to other currently available antibiotics [21]. Recently, Liu et al. reported a novel colistin- resistance gene (mcr-1) was located on the plasmid in Enterobacteriaceae isolated from food-producing animals, retail meat, and humans in China [1]. The gene showed a high transfer rate between *E. coli* strains during conjugation experiments in vitro. Moreover, mcr-2, a variant of mcr-1 and sharing 76.7% nucleotide identity, was revealed soon after the discovery of the paradigm gene mcr-1, which further highlight the prevalence and dissemination of mcr-1 worldwide [22]. MCR-1 is regarded as a phosphoethanolamine transferase via sequence alignment and a member belonging to YhjW/YjdB/YijP superfamily. MCR-1 homologues LptA and EptC can catalyze modification of the 1′ and 4′ phosphoryl groups of lipid A(moiety of lipopolysaccharides) using phosphoethanolamine (PEA) as a substrate to transfer positive charges, which will hinder the interaction and reduce affinity of colistin to lipid A therefore confers colistin resistance to host bacteria [23–25]. Concerning the urgent situation, to search for alternatives to substitute antibiotics and slow down the pace of multi-drug resistance is very essential.

According to many alternatives studied, EOs, with various antimicrobial characteristics, has received significant attention. EOs, as well as their components, has been explored as a source of new antimicrobials on a limited basis. Many EOs were reported to possess significant antimicrobial activity against a wide range of microorganisms [5–7]. Nowadays, EOs continue to be used to treat infectious disease in traditional medicine around the world [15]. Eugenol, the principal chemical ingredient of clove oil has been long known for its local anesthetic, anti-inflammatory, analgesic, antioxidant and antibacterial effects [10, 17]. It belongs to the class of essential oils that is generally recognised as safe (GRAS) by the Food and Drug Administration [26]. Its non-specific permeabilization to the cytoplasmic membrane has been demonstrated in various studies by effluxing potassium and ATP out of the bacterial cells [27–29]. It is thought that the hydroxyl group of eugenol can bind to and affect the properties of proteins and thereby contributing to eugenol’s inhibitory effect at sub-lethal concentrations. Consistent with this, eugenol has proven to inhibit the activity of enzymes including ATPase, histidine decarboxylase, amylase, and protease. Inhibition of the ATPase can result in the destruction of energy generation needed for bacterial cell recovery, and further lead to the death of bacteria [27, 28].

In our study, for most clinical isolated *E. coli* strains, colistin-resistant or colistin-sensitive, eugenol exhibited a synergistic effect on 8 out of 14 cases (FICI, 0.375 to 0.5), while for the rest 6 strains, eugenol exerted partial synergistic effect(FICI, 0.625). In general, MIC for colistin was reduced by 4 to 8-fold in the presence of eugenol. Therefore, we concluded that eugenol could enhance the antibacterial effects of colistin in both colistin-resistant and colistin-sensitive strains. The above results clearly demonstrated that eugenol exhibited strong antibacterial effect and synergistic activity when used alone or combined against colistin-resistant *E. coli* strains. Moreover, we speculate eugenol at a dose of 2 μg/mL may also exhibit a clinical effect when combined with colistin in vitro. However, though eugenol was reported to have a lower haemolytic activity comparing with some therapeutic agent, the dose required to perform in vivo still need further study [5, 30]. In the literature, some studies reported that components with phenolic hydroxyl structures, such as eugenol and thymol are known to possess some antimicrobial activities as bactericidal or bacteriostatic agents against gram-positive and gram-negative bacteria, and their yeasts [5, 31–33]. Moreover, the relative position of hydroxyl group is also crucial for the bioactivity of these components [34].

Multiple drug resistance of *E. coli* is a kind of phenotype. While relative drug resistant genes plays a key role in dominating the phenotype. Studies have indicated that mRNA expression level could restrict degree and stability of multidrug resistance and there existed a positive correlation [35]. Results of real-time PCR clearly showed that expression of mcr-1 gene in synergy group was significantly lower than non-synergy group, which indicated an inhibiting effect of eugenol on mcr-1 gene expression.

Molecular docking is a computational procedure used to predict noncovalent binding of macromolecules or a macromolecule (receptor) and a small molecule (ligand) efficiently, starting with their unbound structures obtained from molecular docking simulations or homology modeling. Docking aim to predict the possible bound conformations and the binding affinity. The prediction of binding of small molecules to proteins is of particular practical importance, which can benefit and be used to screen virtual libraries of drug-like molecules for further drug development [36]. An earlier study have demonstrated better antibacterial activity and predicted eugenol hydrogen bonded with catalytic and other crucial amino acid residues of ESBL proteins of pathogenic bacteria via molecular docking experiments, indicating an effective interactions between them [37]. Thus, to obtain better insight into interactions of eugenol and MCR-1 protein, molecular docking analysis was performed. The results indicated that phenolic hydroxyl group in eugenol could bind with zinc atom of MCR-1 protein in the form of metal ion coordination bond, which could further elucidate phenolic hydroxyl as an important functional group. In addition, catalytic amino acid Ser284, which is in the active pocket of MCR-1 protein was found to hydrogen bond with methoxy in ortho-position of MCR-1 protein, thus stabilizing the docked structures. This is the first study to evaluate antimicrobial property of eugenol against colistin-resistant *E. coli*.

There are also several reports supports that the bioactive components like eugenol, carvacrol and thymol present in EOs could attach to the cell surface, and thereafter, penetrate to the phospholipid bilayer of the cell membrane. Their accumulation disturbs the structural integrity of cell membrane, which will detrimentally influence the cell metabolism and lead to cell death [11, 38]. Similar mode of action was noted by other researchers. EOs, in general, act on cell membrane integrity by changing the membrane permeability which leads to leakage of electrolytes and loss of vital intracellular contents like amino acids, ATP and DNA while inhibiting the energy (ATP) generation and related enzymes leading to the destruction of cell [39–42]. Therefore, we speculate eugenol is likely to interfere with the entire bacterial cell and lead to a cascade of reactions, which act as an inhibitor.

However, the clear mechanism is not completely understood, additional functional studies would be needed to validate the possible antibacterial effect, MCR-1 protein binding and to evaluate their mode of action, which could contribute to the potential efficacy of eugenol to treat clinical colistin-resistant bacteria.

## Conclusions

Our results revealed that eugenol exhibited an synergistic effect with colistin in vitro against a collection of clinical *E. coli* isolates. The synergistic effect might related to the interactions between eugenol and MCR-1 protein.

## Abbreviations

AMP: Ampicillin
AZM: Azithromycin
CFZ: Cefazolin
CTX: Cefotaxime
DMSO: Dimethyl sulfoxide
DOX: Doxycycline
*E.coli*: *Escherichia coli*
EOs: Essential oils
FF: Florfenicol
FICI: Fractional inhibitory concentration index
FRZ: Furazolidone
GEN: Gentamicin
GRAS: Generally recognised as safe
KAN: Kanamycin
LB: Lysogeny broth
LVX: Levofloxacin
MDR: Multidrug-resistance
MHA: Mueller–Hinton agar
MHB: Mueller–Hinton broth
MIC: Minimum inhibitory concentration
NEO: Neomycin
NOR: Norfloxacin
PEA: Phosphoethanolamine
STR: Streptomycin
TCY: Tetracycline
TTC: Triphenyltetrazolium chloride
